# Development of Lightweight Thermoplastic Acrylic PMMA Composites and Characterization of Their Mechanical Properties

**DOI:** 10.3390/polym17111563

**Published:** 2025-06-04

**Authors:** Jiming Sun, Hyeonseok Han, Sooyeon Ahn, Seongsu Jung, Sung Kyu Ha

**Affiliations:** Department of Mechanical Engineering, Hanyang University, 222 Wangsimri-ro, Seongdong-gu, Seoul 04763, Republic of Korea; windymonde12@hanyang.ac.kr (J.S.); hhs4607@hanyang.ac.kr (H.H.); purumi0731@hanyang.ac.kr (S.A.); jumg0520@hanyang.ac.kr (S.J.)

**Keywords:** thermoplastic acrylics, resin infusion process, mechanical property characterization, induction time optimization, glass fiber composites

## Abstract

The effects of benzoyl peroxide (BPO) and dimethylaniline (DMA) composition on the induction time and the tensile strength of thermoplastic acrylic (PMMA) resins have been investigated in this study. Eighteen resin formulations were prepared with different BPO/DMA ratios (2.0–9.5) and DMA contents (0.28–0.65 mol%), and it was observed that tensile strengths reached up to 66 MPa, and induction times (ITs) ranged from 100 to 207 min. Higher BPO/DMA ratios improved tensile strength but shortened IT, while greater DMA content accelerated curing. Polynomial regression models were successfully established, i.e., a third-order equation for the strength and a second-order equation for the IT, based on the BPO/DMA ratio and DMA content to identify the optimal formulation to balance the strength and the IT time. Two selected formulations, P-4-0.5 and P-3-0.3, were applied in vacuum-assisted resin infusion of glass fiber composites. The best-performing unidirectional (UD) laminate achieved a tensile strength of 1244 MPa. As regards ±45° biaxial (BX45) laminates, they exhibited a tensile strength of 124 MPa and a failure strain of 9.02%, which, while lower than that of epoxy, indicates competitive performance. These results demonstrate that the resin was well infused, resulting in 64% higher fiber volume fraction than typical infused glass/epoxy composites, and compositionally optimized PMMA resins can deliver epoxy-comparable strength and enhance damage tolerance in structural composite applications.

## 1. Introduction

As global demand for renewable energy continues to grow, wind power has become a pivotal contributor to achieving sustainability goals. Policies such as the Green New Deal and the 2050 Carbon Neutrality Policy have positioned wind energy as a cornerstone of the hydrogen economy [1]. Wind turbines, particularly their blades, play a vital role in converting wind energy into electricity. However, the increasing deployment of wind turbines has raised significant concerns about end-of-life management for their blades. Typically made of thermosetting resin-based composite materials, wind turbine blades offer high strength and durability but suffer from limited recyclability due to their irreversible crosslinked structure [2]. Once a blade reaches the end of its 20–25 year lifespan, physical degradation or irreparable damage necessitates disposal. Traditionally, disposal methods have included landfill burial and incineration [3]. These methods not only conflict with global carbon neutrality goals but also pose substantial environmental risks. For example, blades in landfills take hundreds of years to decompose, releasing harmful greenhouse gases like methane [4]. While incineration recovers some energy, it produces toxic gases and ash, further exacerbating pollution [5]. By 2050, the global accumulation of blade waste is projected to reach 43 million tons, with 40% generated in China, 25% in Europe, 16% in the United States, and the remaining 19% distributed worldwide [6]. This escalating waste problem highlights the urgent need for alternative solutions, including the development of recyclable materials, advancements in blade design to extend lifespans, and comprehensive lifecycle management policies. Addressing these challenges is crucial to ensuring the long-term sustainability of wind energy as a central pillar of the global renewable energy transition [7].

The growing demand for wind energy has driven the need for advanced manufacturing technologies capable of producing efficient, scalable, and sustainable turbine blades. Among these, resin infusion, originally developed for industries such as wind energy and shipbuilding, has become a widely utilized method for fabricating composite structures. Recent advancements have further highlighted its significance in addressing the challenges of modern wind turbine blade manufacturing. This technique provides distinct advantages that align with the requirements for lightweight yet high-strength composite materials, optimizing energy generation while reducing material usage and environmental impact. For example, using a single-sided mold in conjunction with a vacuum-assisted process significantly reduces tooling costs. Moreover, its compatibility with Automated Fiber Placement (AFP) systems ensures precise fiber alignment and efficient layer stacking, maintaining structural integrity even in large-scale applications. Additionally, the low viscosity of thermoplastic resins, such as MMA-based resins, enhances the process by enabling thorough infiltration into dry fibers without disturbing their alignment. This versatility not only facilitates the production of high-performance blades but also supports the integration of recyclable materials, addressing critical end-of-life waste management challenges in the wind energy sector.

Efforts to address this challenge have driven the development of recyclable resin technologies. Notable examples include recyclable epoxy systems, such as Siemens’s Recyclamine and Vestas’s Swancor, as well as thermoplastic resin systems like Arkema’s Elium, used by LM Wind Power. These innovations enable blades to be chemically or mechanically recycled, overcoming the limitations of traditional thermosetting resins. For instance, Arkema’s Elium resin facilitates the production of thermoplastic composite blades that are lightweight, durable, and recyclable through depolymerization. Similarly, Siemens has introduced recyclable blades using advanced epoxy systems, establishing new benchmarks for sustainability in wind energy [8,9,10]. Furthermore, research on chemical recycling methods has demonstrated significant potential for reclaiming raw materials. Chemical recycling focuses on breaking down polymer chains into monomers, which can be re-polymerized into high-performance resins, whereas mechanical recycling reprocesses materials into secondary products with minimal degradation [11]. The depolymerization of acrylic thermoplastic resins has also emerged as a pivotal technology, offering a scalable approach to recovering high-quality monomers [12]. These advancements not only address the end-of-life challenges of wind turbine blades but also align with global sustainability goals by reducing the environmental impact of composite materials [13]. Recent developments in multifunctional composite systems further highlight the potential of recyclable thermoplastics in structural applications [14].

Arkema’s Elium resin has been a transformative innovation in advancing thermoplastic composite technology for wind turbine blades. Its unique properties—lightweight, high durability, and recyclability through depolymerization—have positioned it as a preferred material in numerous industrial applications. One significant advancement was the fabrication of a 12 m thermoplastic blade by the National Renewable Energy Laboratory (NREL) using Elium resin. This project demonstrated the resin’s compatibility with vacuum-assisted resin transfer molding (VARTM) and its capability to produce large-scale components with exceptional mechanical performance [15]. Furthermore, Arkema’s collaboration with LM Wind Power led to the production of a 100 m thermoplastic composite blade, representing a major milestone in sustainable wind energy solutions [16]. Despite these accomplishments, Elium-based resins for wind turbine blades have certain limitations. The resin’s fixed infusion parameters restrict customization for varying blade lengths or geometries, limiting its adaptability to different manufacturing requirements. Nevertheless, Elium’s successful commercialization has catalyzed global research and innovation in thermoplastic resin systems, underscoring its potential to redefine the future of composite materials [17].

Long before the development of Arkema’s Elium resin, research into acrylic resins established a strong foundation for their industrial applications. Acrylic resins, primarily based on methyl methacrylate (MMA), exhibit exceptional mechanical properties, including high strength, ductility, and impact resistance, while retaining a thermoplastic nature. These attributes, combined with their liquid state, make them ideal for infusion processes used to fabricate complex composite structures [18,19]. The primary constituents of acrylic resins, such as MMA, dimethyl aniline (DMA), and benzoyl peroxide (BPO), have been extensively studied to optimize curing and polymerization behavior. For instance, DMA extends working life and enhances mechanical properties, while BPO effectively initiates polymerization at room temperature [20,21]. Additionally, recent studies on dimethyl terephthalate (DMT) as a resin additive have shown its potential to improve curing kinetics and material toughness under specific conditions [22]. Efforts to enhance acrylic resin performance have also involved incorporating advanced materials like trimethylolpropane trimethacrylate (TMPTMA) to improve thermal stability and mechanical strength. Moreover, the inclusion of nanoparticles such as silica and carbon nanotubes has further enhanced resin properties by improving dispersion and interfacial adhesion, resulting in superior composites for applications in sectors ranging from automotive to renewable energy [23,24]. Extensive research on acrylic resins underscores their versatility and potential to revolutionize composite manufacturing. These advancements not only paved the way for Elium’s commercial success but also highlighted the importance of continuous innovation in acrylic resin technologies.

Despite significant advancements in acrylic resin research, several limitations remain unresolved. Current production methods for PMMA-based acrylic resins are relatively rigid, limiting the ability to tailor resin properties for specific applications. Furthermore, existing techniques for predicting resin infusion, especially for structures with varying lengths or geometries, lack precision and scalability. This limitation hinders the optimization of the infusion process for large-scale applications, such as wind turbine blades [25]. Additionally, a comprehensive modeling approach that links resin composition to key factors, such as induction time and mechanical properties, is largely absent in current research [26].

A critical challenge lies in optimizing induction time and tensile strength, which are essential for large-scale composite structures like wind turbine blades. Induction time plays a pivotal role in the resin infusion process, ensuring sufficient working time before curing begins. During the infusion of large-scale structures, premature curing can lead to incomplete infiltration, resulting in voids or defects in the composite. Optimizing induction time is therefore vital to balancing process efficiency with complete resin distribution, ensuring structural integrity [24]. Tensile strength, on the other hand, is a key performance indicator for assessing the mechanical performance of composite materials. High tensile strength ensures that the composite can withstand operational loads and stress, making it a key parameter for evaluating material performance [12].

Despite these considerations, current research lacks a systematic methodology for optimizing resin performance tailored to large-scale wind turbine blade applications. Existing studies often fail to establish correlations between critical parameters such as induction time, viscosity, and mechanical strength, making it challenging to achieve consistent manufacturing performance [25]. Moreover, comprehensive evaluations of mechanical properties, including tensile strength and fatigue performance, remain scarce [26].

To address these challenges, this study proposes a methodology for developing recyclable thermoplastic resin systems optimized for large-scale structural applications. By establishing predictive models to optimize resin composition and conducting detailed evaluations of induction time and tensile strength, this research provides a reliable framework for manufacturing sustainable wind turbine blades. Additionally, the study validates these methodologies in composite materials, facilitating direct comparisons between experimental and simulated conditions and addressing real-world challenges in resin infusion and curing processes [12,24].

## 2. Methodology

### 2.1. Materials

**Figure 1:**
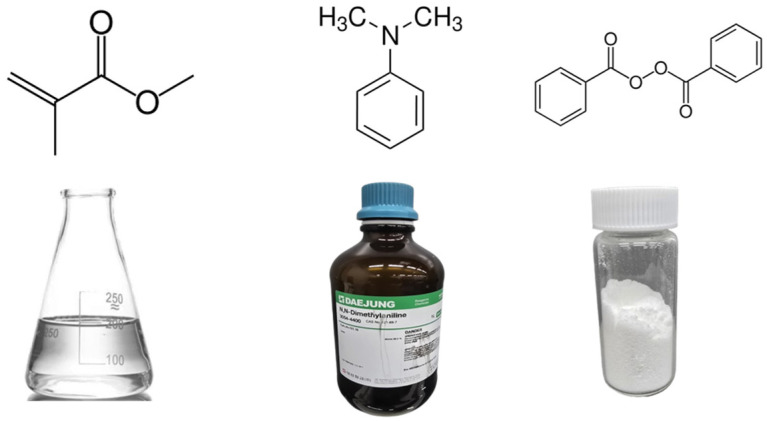
Chemical formulas and sample photos of components required for polymerization.

The materials used in this study include methyl methacrylate (MMA), benzoyl peroxide (BPO), and dimethyl aniline (DMA) (Figure 1). MMA was sourced from LX MMA, Seoul, Republic of Korea, while DMA was purchased from Duksan General Science, Ansan, Republic of Korea. All materials were used without further purification. MMA was chosen as the primary monomer for its superior mechanical properties and recyclability potential. BPO served as the curing agent, initiating radical polymerization and significantly affecting both the curing speed and the final resin properties. DMA was utilized as an accelerator to regulate the polymerization reaction between MMA and BPO.

The use of DMA as an accelerator in MMA polymerization is well documented for its ability to enhance polymerization rates and improve material properties. DMA stabilizes the free radicals generated during the initiation stage, influencing polymerization kinetics and controlling the curing process. This interaction results in improved mechanical properties and increased crosslink density in the final resin, which are critical for applications requiring durable composite materials. Previous studies have demonstrated the effectiveness of DMA in regulating polymerization reactions and achieving consistent curing outcomes.

In related studies, Suzuki et al. [11] investigated the kinetics and temperature evolution during the bulk polymerization of MMA, focusing on the effects of initiators like BPO and accelerators such as DMA. Their findings emphasized the temperature-dependent behavior of the polymerization process, which significantly impacts curing speed and final material properties. Gong et al. [26] examined the influence of initiator content on the room temperature polymerization of MMA, showing how variations in initiator concentration affect polymerization rates. This research provides valuable insights into optimizing curing speed and resin properties. Similarly, Przesławski et al. [23] studied the effects of different initiator concentrations on the polymerization kinetics of methacrylate bone cement, confirming the critical role of initiator concentration in determining polymerization behavior and resulting material properties.

Achilias and Sideridou [11] explored the interaction between BPO/amine initiation systems and the free-radical polymerization of MMA, highlighting the importance of the initiator–accelerator relationship in achieving desired material properties, particularly for dental resins and bone cements. Additionally, Suzuki et al. [25] investigated phase separation behavior during the bulk polymerization of MMA, focusing on the interaction between the initiator, monomer, and accelerator. Their study revealed that phase separation during polymerization significantly influences the final morphology and mechanical properties of the resin, a finding highly relevant to the current research. Understanding phase separation behavior and polymerization kinetics provides essential guidance for optimizing curing processes and material properties in this study.

### 2.2. Resin Curing Process

The resin curing process began with the preparation of the resin mixture. DMA was first added as the accelerator, followed by MMA, and finally BPO as the curing agent. The components were mixed at room temperature for less than 1 min using a magnetic stirrer to ensure a homogeneous mixture. Once the resin mixture was uniform, the curing process proceeded in two stages.

The primary curing stage was conducted at room temperature, allowing the resin to partially set and form an initial structure. This was followed by secondary curing in a convection oven at 70 °C for 2 h, ensuring that the resin achieved its final hardness and stability by completing the polymerization reaction. After curing, the specimens were gradually cooled to room temperature to prevent thermal shock that could compromise the final material properties.

Once cooled, the specimens were carefully removed from the molds to prevent damage to the consolidated material. The surfaces were then sanded to achieve a smooth and uniform finish, which was essential for subsequent mechanical testing. This multi-stage curing process was specifically designed to optimize the material’s properties, ensuring that the resin attained the desired mechanical strength and durability.

### 2.3. Induction Time Measurement

Induction time is a critical parameter for understanding the curing behavior of resin systems, particularly in bulk polymerization processes. It is defined as the time interval from the initiation of the polymerization reaction to the point where the curvature of the temperature–time curve reaches its maximum value. This point signifies the onset of the Trommsdorff effect, a phenomenon characterized by rapid acceleration in the polymerization reaction rate. This acceleration occurs due to a significant increase in viscosity, which inhibits chain termination while allowing continued chain propagation. According to Suzuki et al. [25], the Trommsdorff effect is driven by diffusion-controlled dynamics of macroradicals and monomers, resulting in a self-accelerating reaction process that profoundly influences the temperature profile and overall curing behavior.

To measure the induction time, this study followed experimental protocols similar to those reported by Suzuki et al. [25] and Gong et al. [26]. Specifically, 10 g of the resin mixture (MMA, BPO, and DMA) was placed into a 20 mL vial, as described in these studies. A hole was drilled into the vial lid, and a K-type thermocouple was inserted to monitor the internal temperature. The lid was sealed with vacuum sealant tape to minimize external temperature interference and environmental influences.

**Figure 2:**
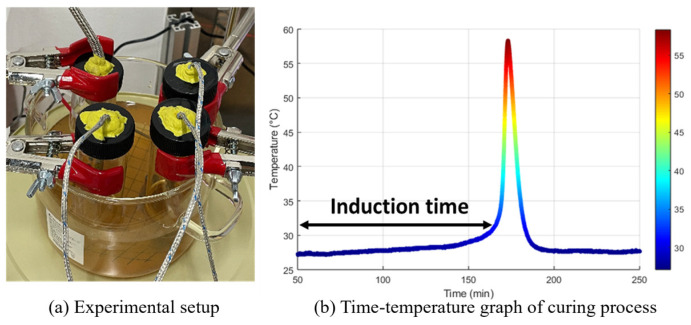
(**a**) Experimental setup to measure the temperature evolution during the curing reaction of resin in constant temperature bath; (**b**) time–temperature graph of curing resin process.

The prepared vial containing the resin sample was immersed in a constant-temperature water bath maintained at 25–26 °C to ensure stable experimental conditions. During curing, the exothermic reaction was recorded, and the induction time was determined from the temperature profile. As shown in Figure 2, the induction time corresponds to the interval preceding a significant temperature gradient change, which correlates with the onset of the Trommsdorff effect. This method, based on established protocols [25,26], allowed precise monitoring and characterization of the curing behavior of the resin system.

### 2.4. Resin Specimen Preparation and Tensile Testing

**Figure 3:**
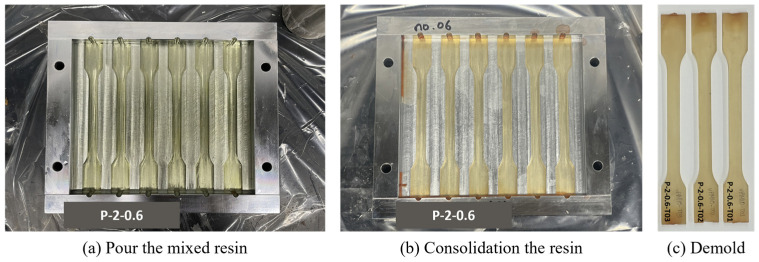
(**a**) Pour the mixed resin into ISO 527-2 steel mold, after applied release agent. (**b**) Consolidation at room temperature, followed by post curing (70 °C for 2 h). (**c**) Demold and sand lightly to make the surface uniform.

Tensile test specimens were prepared in accordance with ISO 527-2 standards using steel molds, as illustrated in Figure 3 [27]. The molds were coated with a mold release agent, which was applied four times at 30 min intervals and thoroughly cleaned to ensure easy demolding and prevent surface defects. The resin mixture was poured into the molds, and a polypropylene film was placed over the top to minimize the evaporation of volatile components during curing.

Once the curing process was completed, the specimens were carefully demolded and prepared for testing. Tensile tests were performed using a Universal Testing Machine (UTM) equipped with an extensometer to ensure precise strain measurements. A speed rate of 1 mm/min was applied, and stress–strain curves were recorded to determine key mechanical properties, including ultimate tensile strength and Young’s modulus. The complete process, from mold preparation to final specimen testing, is depicted in Figure 3.

## 3. Polymerization Cases of MMA with Various BPO and DMA Concentrations

### 3.1. Experimental Design and Variable Selection

To generate response models for tensile strength and induction time, we selected 18 resin cases by varying the concentrations of benzoyl peroxide (BPO) and dimethylaniline (DMA). Given the chemical importance of their interaction, the BPO/DMA (B/D) ratio and DMA molar content were defined as independent experimental variables. The B/D ratio effectively represents the balance between the initiator and the accelerator, which is critical for understanding curing kinetics. This formulation-based approach enables the analysis of key processing–performance relationships, with relevance to composite manufacturing applications requiring both mechanical performance and process efficiency.

**Figure 4:**
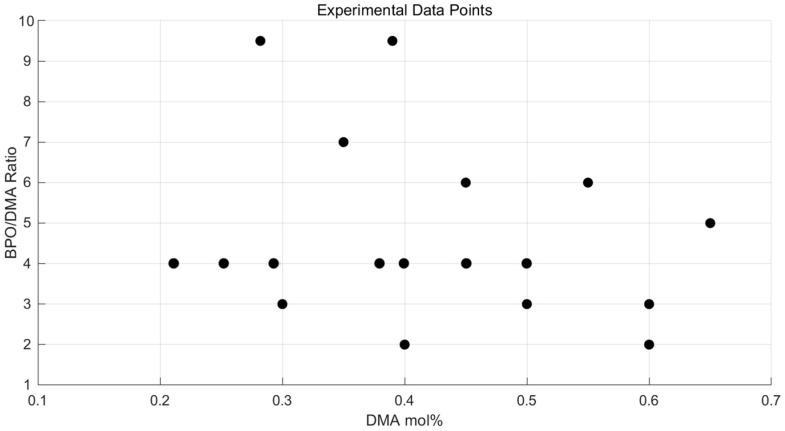
Experimental data points test showing 18 cases with varying B/D ratios (2.0–9.5) and DMA mol% values (0.28–0.65).

A total of 18 cases were tested, with B/D ratios ranging from 2.0 to 9.5 and DMA contents from 0.28 to 0.65 mol%. The test cases are presented in Figure 4 and Table 1, with each case labeled as P-x-y, where x and y represent the B/D ratio and DMA mol%, respectively. To investigate the relationship between tensile strength and induction time, the B/D ratio was varied starting from 2, which is the minimum ratio required to initiate a stable reaction between BPO and DMA [26].

### 3.2. Measurement of Induction Time

**Figure 5:**
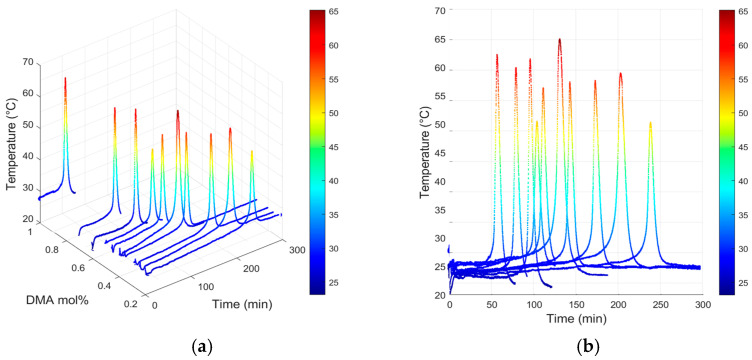
(**a**) 3D plot of induction time—B/D ratio = 4; (**b**) 2D plot of induction time—B/D ratio = 4.

The consolidation behavior of the resin formulations was further analyzed to examine the effect of DMA concentration on induction time at a fixed B/D ratio of 4. Figure 5 shows the temperature–time profiles obtained from experimental measurements, presented in both 3D and 2D visualizations. These profiles illustrate the dynamic temperature evolution during the curing process and provide insight into the exothermic characteristics of the resin formulations.

The temperature–time profiles reveal a clear relationship between DMA mol% and induction time. Higher DMA concentrations led to an earlier onset of the Trommsdorff effect, as indicated by a significant temperature rise within a shorter time. In contrast, lower DMA concentrations resulted in delayed polymerization, characterized by extended induction times. This trend highlights the critical role of DMA concentration in influencing curing dynamics and the overall exothermic behavior of the resin system.

The 3D plot in Figure 5 visually represents the temperature distribution over time for various DMA mol% values. The 2D plot provides a clear visualization of the time interval before the Trommsdorff effect, enabling a detailed analysis of induction time changes. These findings demonstrate that higher DMA concentrations enhance the interaction between the curing agent and the accelerator, thereby accelerating the curing reaction. The experimental results align with theoretical predictions, confirming the validity of the proposed framework.

**Figure 6:**
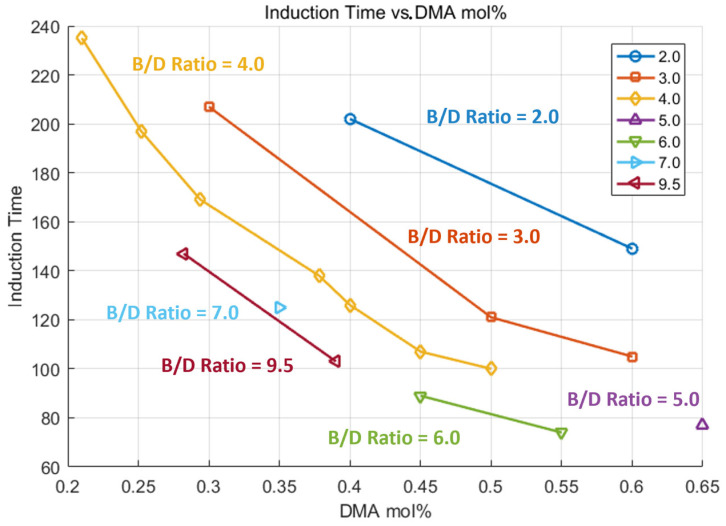
Grouped by B/D ratio, experiment result of induction time—DMA mol%.

Increasing DMA mol% leads to a reduction in induction time across all B/D ratios, as shown in Figure 6. This trend reflects the accelerating effect of DMA on the polymerization reaction. It should be noted that, for large-scale structures like wind turbine blades, a longer induction time is desirable to ensure sufficient working time for resin infiltration before curing begins. Selecting formulations with moderate DMA content and lower B/D ratio helps maintain a longer induction time while achieving the optimal strengths, which is critical for achieving complete fiber wet-out and minimizing voids during infusion.

### 3.3. Measurement of Strengths

To further explore how resin composition affects mechanical performance, systematic experiments were conducted to evaluate the influence of B/D ratio and DMA mol% on tensile strength. The goal was to identify optimal combinations that deliver high strength while maintaining sufficient working time during curing.

**Figure 7:**
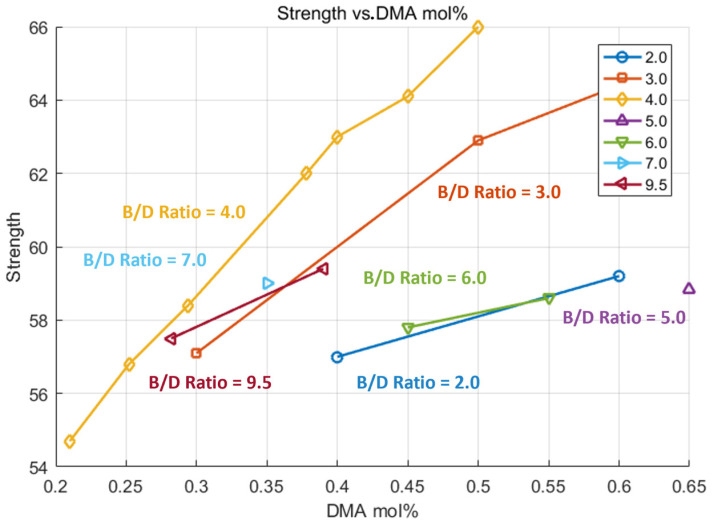
Grouped by B/D ratio, experiment result of strength—DMA mol%.

Tensile strength increases significantly with higher DMA mol%, particularly when the B/D ratio is 4 or lower, as shown in Figure 7. However, the rate of improvement tapers off at a higher ratio, indicating a non-linear relationship. While higher DMA levels enhance strength, they also reduce induction time (as discussed previously in Figure 6), which can limit processability for large composite structures. Therefore, a balanced formulation—such as a DMA of around 0.5 mol% with a B/D ratio near 4—offers an effective compromise between mechanical performance and manageable curing behavior.

**Figure 8:**
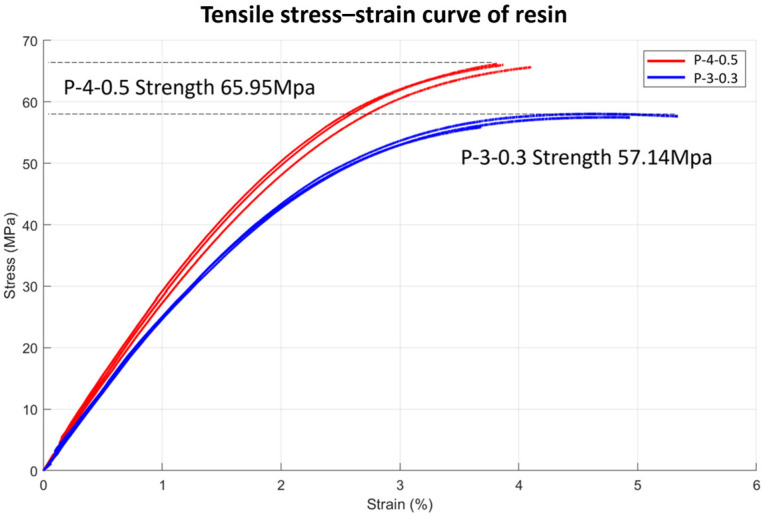
Tensile stress–strain curve of resin, ‘P-4-0.5’ and ‘P-3-0.3’.

Building on this analysis, Figure 8 presents the stress–strain curves obtained from tensile tests of the resins, providing insights into the relationship between induction time (*IT*) and mechanical behavior. P-4-0.5 demonstrated higher tensile strength with a shorter *IT* of 100 min, whereas P-3-0.3 exhibited lower tensile strength but a substantially longer *IT* of 207 min. This trade-off underscores the inverse relationship between *IT* and tensile strength, demonstrating how resin composition governs mechanical performance and curing behavior. Shorter *IT* values enhance tensile strength but may lead to brittleness, as indicated by reduced failure strain. In contrast, longer *IT* values improve ductility while reducing tensile strength, as observed in P-3-0.3. These results emphasize the importance of balancing *IT* and mechanical properties to optimize composite performance.

The observed experimental trends validate the effectiveness of regression models in predicting mechanical properties and curing behavior based on compositional parameters. These models provide a robust framework for understanding the interplay between tensile strength and induction time, which will be further detailed in the subsequent section.

## 4. Predictive Modeling for Resin Optimization

### 4.1. Quadratic Regression Model

A general polynomial regression model was developed to analyze the relationships between tensile strength and induction time with respect to DMA mol% and the B/D ratio. These models were designed to capture the non-linear interactions between the two variables and provide a deeper understanding of the curing behavior of the resin system in relation to its mechanical properties.

Polynomial equations were employed to represent the observed data trends. For the tensile strength (*S*) model, second-order terms for DMA mol% (*x*) and third-order terms for the B/D ratio (*y*) were used to account for variable interactions, as shown in Equation (1). Similarly, the induction time (*IT*) model utilized second-order terms for both DMA mol% (*x*) and the B/D ratio (*y*), as shown in Equation (2).

(1) $$S=p_{00}+p_{10}x+p_{01}y+{p_{20}x}^{2}+p_{11}xy+p_{02}y^{2}+p_{21}x^{2}y+p_{12}{xy}^{2}+p_{03}y^{3}$$

(2) $$IT=p_{00}+p_{10}x+p_{01}y+{p_{20}x}^{2}+p_{11}xy+p_{02}y^{2}$$

Polynomial coefficients for strength and *IT* regression models were determined using MATLAB’s Polyfit method [28] with the least-squares approach. These models effectively capture the non-linear relationships between DMA mol%, B/D ratio, and their effects on strengths and *IT*. The constant coefficients derived from the regression models are listed in Table 2 and show strong agreement with experimental data.

**Figure 9:**
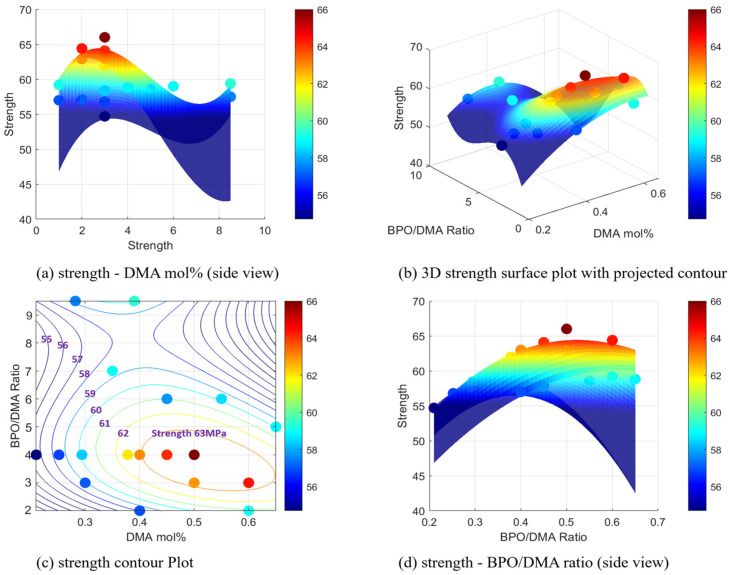
Strength surface and contour plots with experimental points, showing the effects of DMA mol% and B/D ratio: (**a**) strength—B/D ratio (Side view), (**b**) 3D strength surface plot with projected contour, (**c**) strength contour plot, and (**d**) strength—DMA mol% (side view).

The variation in tensile strength with respect to DMA mol% and the B/D ratio is illustrated in Figure 9. The 3D surface plot (Figure 9b) highlights the non-linear interaction between these two variables, while the contour plot (Figure 9c) provides a 2D representation that helps identify regions of high tensile strength. Strength generally increases with the B/D ratio, peaking around a value of 3, and then declines at higher ratios. Similarly, optimal tensile strength is observed when DMA mol% falls within the range of approximately 0.3 to 0.6. These findings emphasize the importance of appropriately balancing the curing agent and accelerator to achieve superior mechanical performance.

**Figure 10:**
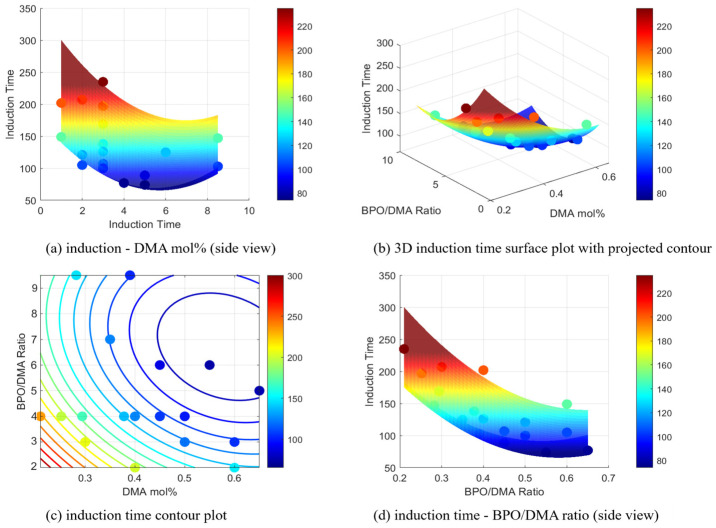
Induction time surface and contour plots with experimental points, showing the effects of DMA mol% and B/D ratio: (**a**) induction time—B/D ratio (side view), (**b**) 3D induction time plot with projected contour, (**c**) induction time contour plot, and (**d**) induction time—DMA mol% (side view).

The corresponding effects on *IT* are shown in Figure 10. The 3D surface plot (Figure 10b) demonstrates that *IT* decreases as both DMA mol% and the B/D ratio increase. This trend is further supported by the contour plot (Figure 10c), which shows that the shortest *IT*s occur at B/D ratios above 4 and DMA mol% between 0.2 and 0.5. This behavior indicates a more efficient curing process driven by the enhanced interaction between the initiator and the accelerator under these conditions.

Taken together, the analysis of tensile strength and *IT* provides a comprehensive understanding of how resin composition influences both mechanical and curing behavior. The overlapping regions where tensile strength is maximized and *IT* is minimized suggest that optimal performance can be achieved with a B/D ratio between 3 and 4 and a DMA concentration between 0.3 and 0.5 mol%. This range represents a robust formulation window for balancing curing efficiency and mechanical properties in PMMA resin systems.

### 4.2. Prediction of Maximum Strength for a Given Induction Time Limit

This analysis seeks to determine the maximum achievable tensile strength for a given *IT* constraint using the polynomial regression models developed earlier. By overlaying the regression outputs for strength and *IT* on a single contour plot, intersections between strength contours and *IT* contours were identified. These intersection points indicate optimal combinations of DMA mol% and B/D ratio that achieve the highest mechanical performance while satisfying specified processing constraints.

During the fabrication of PMMA resin specimens, excessive bubble formation was observed when the *IT* fell below 100 min. To reflect realistic manufacturing requirements, particularly for large-scale wind turbine blade production, the *IT* range in this analysis was set between 100 and 260 min. This range ensures a practical balance between rapid curing and high-quality composite fabrication.

**Figure 11:**
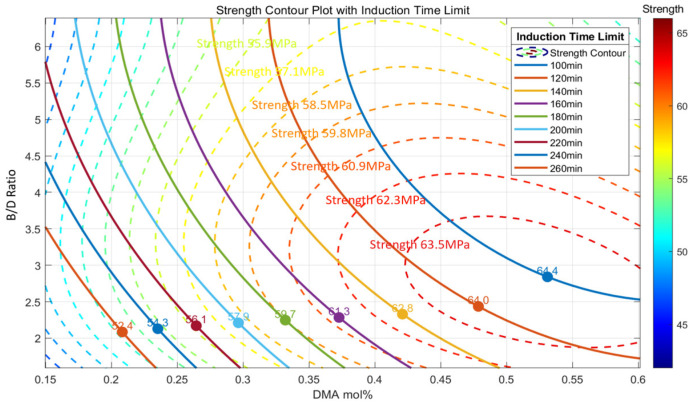
Strength contour plot with induction time limit.

As shown in Figure 11, the solid lines represent *IT* contours, while the dashed lines correspond to tensile strength. The combined visualization highlights regions where high strength aligns with acceptable curing windows. The results indicate that shorter *IT*s within the allowable range are generally associated with higher tensile strength. These findings underscore the importance of optimizing resin formulation to balance curing kinetics and mechanical performance effectively.

**Figure 12:**
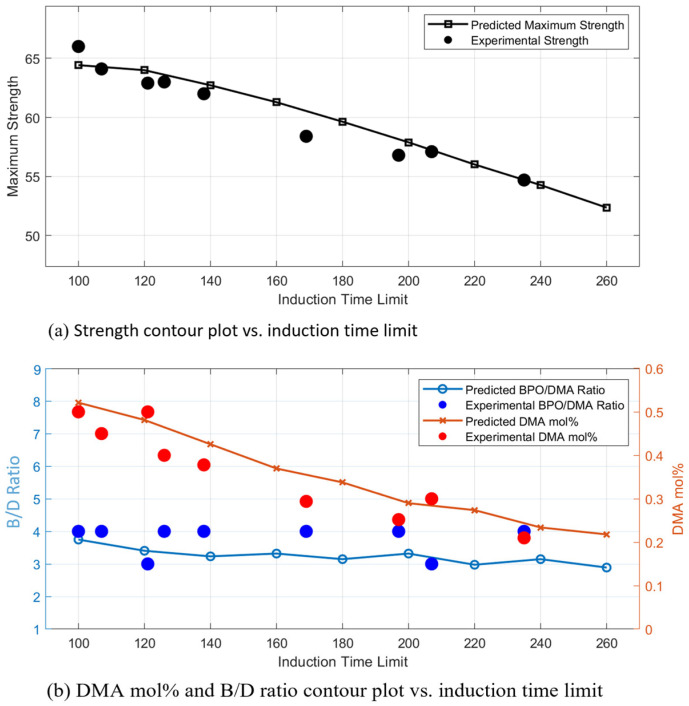
Predicts maximum strength and composition ratios (BPO/DMA ratio, DMA mol%) based on regression models; (**a**) strength contour plot vs induction time limit; (**b**) DMA model% and B/D contour plot vs. induction time limit.

The predicted maximum tensile strength, corresponding B/D ratio, and DMA mol% at various *IT* limits were derived from the regression models presented in Figure 11 and are visualized as a 3D surface plot in Figure 12. These predictions were obtained by identifying the composition conditions that yield peak strength across a range of allowable *IT*s.

The model enables the estimation of the optimal formulation, i.e., the combination of B/D ratio and DMA mol%, required to achieve maximum strength for any specified *IT* constraint. To validate the model, predicted strength values were compared with experimental results within a ±5% error margin. The associated composition ratios from these experiments were then cross-checked against the predicted values.

The comparison showed strong agreement between experimental and predicted formulations, supporting the reliability and accuracy of the regression framework. These findings confirm that the model offers a reliable and objective method for optimizing resin formulations based on curing time constraints.

## 5. Validation Through Composite Testing

### 5.1. Composite Panel Fabrication

The composite manufacturing process involved resin infusion into unidirectional glass fiber fabrics to produce high-performance composite panels. The unidirectional glass fiber used was OC-L1240 from Owens Corning. Each panel consisted of two layers of 400 × 400 mm glass fiber arranged at 0° orientation to ensure uniform reinforcement distribution within the composite structure.

**Figure 13:**
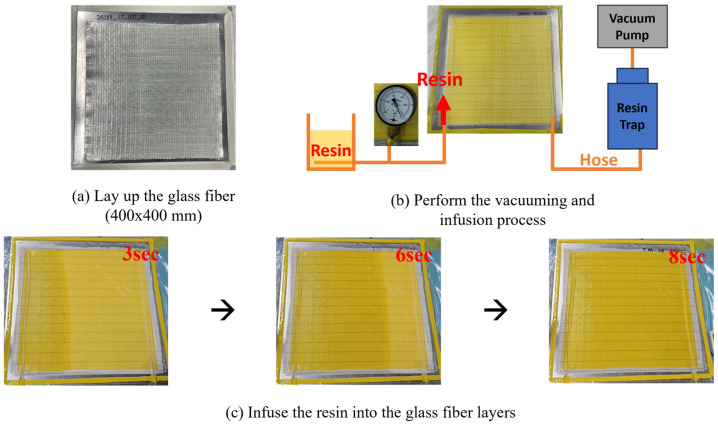
(**a**) Apply release agent and sealant tape, layup glass fiber, 2-layer 400 × 400 (mm) glass fiber, 0° orientation, OC-L1240. (**b**) Lay up the infusion consumables, and vacuuming for at least 15 min. (**c**) Infuse the resin into the glass fiber layers.

Two resin formulations, P-4-0.5 and P-3-0.3, were selected for the infusion process. Prior to resin infusion, the molds were prepared by applying a mold release agent four times at 30 min intervals to ensure smooth demolding and prevent surface defects. After surface treatment, a peel ply and a flow mesh were sequentially placed over the glass fiber layers to enhance resin flow during infusion. Spiral tubes and vacuum hoses were positioned to ensure uniform resin distribution, as shown in Figure 13.

To initiate the infusion process, the vacuum bag was sealed, and a vacuum was applied for at least 15 min to establish a stable vacuum environment. The resin was then introduced under vacuum pressure, allowing it to fully infiltrate the fiber network. This method ensured thorough wetting of the reinforcement and uniform consolidation of the composite panel.

Following the infusion, the composite panels underwent a two-stage curing process. The primary curing was conducted at room temperature to partially solidify the resin. Subsequently, secondary curing was carried out in a convection oven at 70 °C for 2 h. After curing, the consolidated composites were gradually cooled to room temperature to prevent internal stress and ensure structural integrity. The consolidated composite panels were cut into tensile test specimens according to ASTM D3039 standards [29].

### 5.2. Induction Time Evaluation in Composite Panels

To compare the *IT* behavior observed in vial-scale experiments with that of 400 × 400 mm composite panels, thermocouples were used to monitor temperature during the curing process. Composite panels fabricated with the resin formulations P-4-0.5 and P-3-0.3 were prepared using ±45° biaxial glass fiber reinforcement and infused under vacuum.

**Figure 14:**
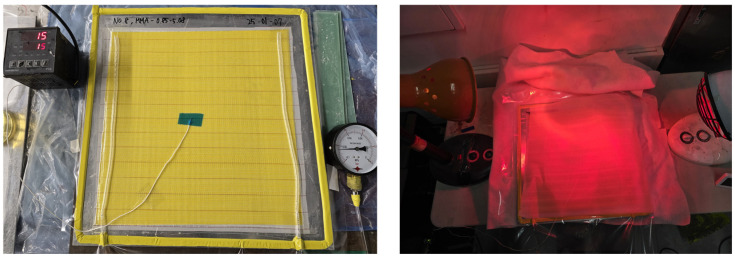
Measurement process of induction time during the composite curing process.

To reduce heat loss during curing, the panels were wrapped in insulating cloth, as illustrated in Figure 14. Temperature measurements were initiated immediately after infusion, with thermocouples placed at the geometric center of each panel to accurately capture the internal thermal response.

**Figure 15:**
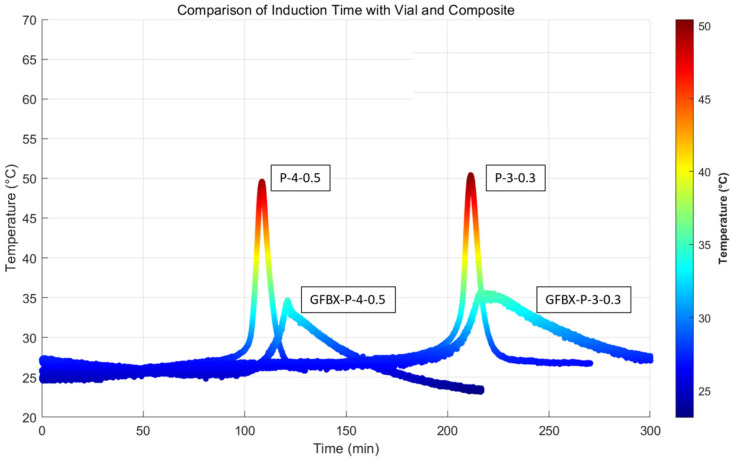
Comparison of induction time with vial and composite.

The temperature–time profiles obtained from 10 mL vials and composite panels are compared in Figure 15. Although the maximum temperature observed in the composite panels was lower than that in the vial primarily due to heat loss during curing, the measured *IT*s were remarkably similar between the two setups.

This result suggests that the simplified vial-based experiments effectively replicate the *IT* behavior observed in actual composite curing, despite thermal differences. Consequently, the vial method can be considered a reliable and practical approach for predicting *IT* under real processing conditions.

### 5.3. Tensile Tests of Glass/PMMA Composites

**Figure 16:**
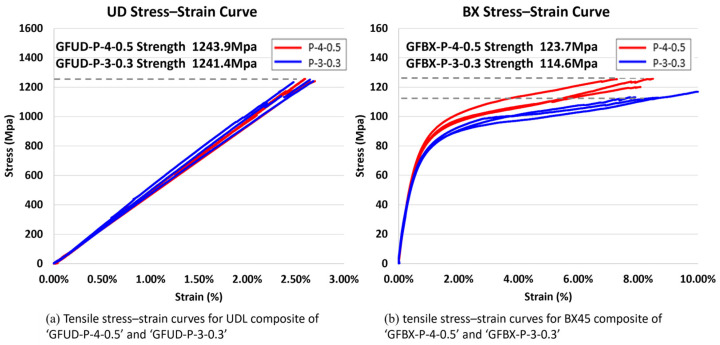
(**a**) Tensile stress–strain curves for UDL composite of ‘GFUD-P-4-0.5’ and ‘GFUD-P-3-0.3’; (**b**) tensile stress–strain curves for BX45 composite of ‘GFBX-P-4-0.5’ and ‘GFBX-P-3-0.3’.

Tensile tests were performed to evaluate the relationship between resin properties and composite mechanical performance. Tests were conducted on unidirectional laminates (UDLs) and ±45° biaxial laminates (BX45) fabricated using the P-3-0.3 and P-4-0.5 resin systems. All specimens were prepared in accordance with ASTM D3039, and tensile loading was applied at a crosshead speed of 1 mm/min. The stress–strain responses are presented in Figure 16, and the corresponding mechanical properties are summarized in Table 3.

As with the resin itself, we observed a similar trend in the composites: shorter *IT*s generally resulted in higher tensile strength. In the glass fiber unidirectional laminate (GFUD) configuration, composites fabricated with the two PMMA formulations (GFUD-P-3-0.3 and GFUD-P-4-0.5) exhibited nearly identical tensile strength values of 1241 MPa and 1244 MPa, respectively. This suggests that mechanical performance under fiber-dominated loading is relatively insensitive to differences in resin formulation. However, compared with the epoxy reference used in wind turbine blades, which typically exhibits a tensile strength of 972 MPa [30], the GFUD/PMMA composites showed roughly 27% higher tensile strength. This improvement is primarily attributed to their higher fiber volume fraction (64% vs. 54%), which was achieved due to the lower viscosity of the PMMA resin during the infusion process. The increased fiber content plays a dominant role in enhancing mechanical performance under unidirectional loading conditions.

In contrast with the unidirectional configuration, the ±45° biaxial laminate (BX45) configuration revealed a stronger dependence on resin formulation. The GFBX-P-4-0.5 composite exhibited higher tensile strength (124 MPa) than GFBX-P-3-0.3 (115 MPa), although both values were lower than that of the epoxy-based composite (143 MPa) [30]. The tensile behaviors of ±45° laminates, commonly used to evaluate in-plane shear resistance, were compared between PMMA and epoxy composites to assess matrix-dominated performance. Therefore, we compared the tensile behaviors of ±45° laminates between PMMA and epoxy composites to evaluate their relative shear performance. This comparative assessment is reflected in the data and discussion.

In contrast with the unidirectional configuration, the ±45° biaxial laminate (BX45) configuration revealed a stronger dependence on resin formulation. The GFBX-P-4-0.5 composite exhibited higher tensile strength (124 MPa) than GFBX-P-3-0.3 (115 MPa), reflecting the dominant role of matrix properties under shear-dominated loading, as also observed in the neat PMMA resins. The failure strains of the PMMA-based BX45 composites were lower than those of the epoxy reference: GFBX-P-3-0.3 and GFBX-P-4-0.5 exhibited failure strains of 9.02% and 7.96%, respectively, while some epoxy-based composites exceeded 10% [31]. These differences in mechanical performance can be attributed to the PMMA resin being relatively softer and less strong than epoxy.

## 6. Summary and Conclusions

This study developed polynomial regression models to predict both induction time (*IT*) and tensile strength based on two key composition parameters: DMA mol% and BPO/DMA (B/D) ratio. These models captured the complex interplay among MMA, DMA, and BPO, enabling an effective formulation strategy that effectively balances processing time with mechanical performance. The predictive framework provides a reliable method for identifying optimal resin compositions to achieve maximum tensile strength within a target *IT* range.

Unidirectional tensile strength, among other properties, was selected to validate the performance of the resin in the composites. This is consistent with the requirements for spar caps and stiffening elements in wind turbine blades, where the axial tensile stiffness and strength are the dominant design factors. These formulations were implemented in composite panel fabrication via VARTM. The resins exhibited excellent infusion characteristics, enabling uniform impregnation of both unidirectional (UD) and biaxial (BX) fiber reinforcements. Compared with conventional epoxy systems, the composites achieved higher fiber volume fractions, demonstrating improved flowability and fiber packing. The fabricated panels also exhibited stable mechanical behavior, indicating that PMMA resins are well suited for producing recyclable, high-performance composite structures using standard infusion techniques.

It can be concluded that the PMMA resins demonstrate strong potential to replace conventional thermosetting resins in large-scale structural infusion processes. Their tunable processing behavior, combined with competitive mechanical properties and inherent recyclability, offers significant advantages in terms of design flexibility and alignment with sustainability and circular economy goals.

However, fatigue behavior, moisture resistance, and thermal moisture aging need to be deeply studied for applications such as large wind turbine blades. The debonding phenomena due to fiber–matrix interfacial behavior, also need to be further examined. Fiber sizing tailored for PMMA resins may play a critical role in enhancing long-term composite performance.

To commercialize the current PMMA resin as a new resin system, a cost analysis also needs to be performed to be competitive to the conventional resin system. To commercialize the current PMMA resin as a new resin system, a cost analysis also needs to be performed to be competitive to the conventional resin system. The applicability of PMMA-based composites can be extended to fatigue-critical applications, where their performance under cyclic loading may support long-term use in high-efficiency, recyclable composite structures.

## Figures and Tables

**Table 1:** Experimental data of DMA mol%, B/D ratio, tensile strength, and induction time (P-x-y, where x = B/D ratio and y = DMA mol%).

Test ID: P-x-y	P-2-0.4	P-2-0.6	P-3-0.3	P-3-0.5	P-3-0.6	P-4-0.21	P-4-0.25	P-4-0.29	P-4-0.38	P-4-0.4	P-4-0.45	P-4-0.5	P-5-0.65	P-6-0.45	P-6-0.55	P-7-0.35	P-9.5-0.28	P-9.5-0.39
B/D Ratio, x	2	2	3	3	3	4	4	4	4	4	4	4	5	6	6	7	9.5	9.5
DMA (mol%), y	0.4	0.6	0.3	0.5	0.6	0.21	0.25	0.29	0.38	0.4	0.45	0.5	0.65	0.45	0.55	0.35	0.28	0.39

**Table 2:** Coefficients of Polynomial Equations (1) and (2) for strength and *IT*.

	*p_00_*	*p_10_*	*p_01_*	*p_20_*	*p_11_*	*p_02_*	*p_21_*	*p_12_*	*p_03_*
Strength, *S*	15.68	107.99	11.46	−65.25	8.51	−2.76	−15.36	−0.32	0.19
Induction Time, *IT*	341.46	1507.63	−54.04	836.73	23.78	3.52	0	0	0

**Table 3:** Comparative data of P-3-0.3, P-4-0.5, epoxy performance in UDL and BX45 composites (UDL: unidirectional laminate; BX45: ±45° biaxial laminate).

	UDL			BX45			
	P-3-0.3	P-4-0.5	Epoxy [30]	P-3-0.3	P-4-0.5	Epoxy [30]	Epoxy [31]
Volume Fraction	64%	64%	54%	64%	64%	44%	-
Stiffness (Gpa)	46.9	46.6	41.8	13.1	14.2	13.6	8.2
Strength (Mpa)	1241	1243.9	972	115	124	144	78.7
Failure strain (%)	2.65	2.65	2.44	9.02	7.96	2.16	>10

## Data Availability

The original contributions presented in this study are included in the article. Further inquiries can be directed at the corresponding author.
